# International Perspectives Concerning Donor Milk Banking During the SARS-CoV-2 (COVID-19) Pandemic

**DOI:** 10.1177/0890334420917661

**Published:** 2020-03-30

**Authors:** Kathleen A. Marinelli

**Affiliations:** 1 12227 Department of Pediatrics, University of Connecticut School of Medicine, Division of Neonatology, Connecticut Children’s’ Medical Center, Hartford, CT, USA

**Keywords:** breastfeeding, Coronavirus 2, donor milk, donor milk banking, European Milk Banking Association, Severe Acute Respiratory Syndrome Coronavirus-2, COVID-19, Human Milk Banking Association of North America

## Background

On December 31, 2019 the first case of what is now known as severe acute respiratory syndrome coronavirus 2 (SARS-CoV-2) was reported to the Chinese Center for Disease Control and Prevention. In late December 2019, in Wuhan, Hubei Province, China, clusters of patients with pneumonia of unknown cause, linked to a seafood and animal wholesale market there, began to surface. By January 3, 2020, 44 cases were reported in China. Chinese authorities isolated a novel coronavirus, on January 7, 2020, and shared its genetic sequence on January 12. It was identified in Thailand on January 13, Japan on January 15, and January 20 in Korea. Deaths were already being reported in China. The first case in the United States was reported on January 21, a U.S. citizen who had been in Wuhan. Human to human transmission was suggested by January 24, and by the 25th it had spread to Australia and France. By January 27 it was confirmed in 11 countries outside of China. On January 30, the World Health Organization (WHO) announced that the COVID-19 outbreak was a Public Health Emergency of International Concern. The first two cases were reported in Italy on January 31 (World Health Organization, 2020a). The spread continued, and on March 11 WHO characterized COVID-19 as a pandemic, acknowledging the disease’s geographical spread. On March 16, the total number of cases and deaths outside China surpassed the totals in China. On March 24, 2020, the date of this writing, there are 395,647 confirmed cases and 17,240 deaths worldwide (Johns Hopkins University Medicine, 2020). These numbers will certainly be higher at publication.

The International Committee on Taxonomy of Viruses (ICTV) announced the name of this novel coronavirus as “severe acute respiratory syndrome coronavirus 2 (SARS-CoV-2)” on February 11. This name was chosen because the virus is genetically related, but different, to the coronavirus responsible for the Severe Acute Respiratory Syndrome (SARS) outbreak in 2003 (World Health Organization, 2020b). WHO shortened the name coronavirus disease 2019 to “COVID-19.”

In addition to the concerns for the general public, there are heightened concerns for the more at risk populations: the elderly, those with comorbidities, and the immunosuppressed. Another population of concern is pregnant women. The other two well-known coronaviruses, severe acute respiratory syndrome (SARS-CoV) and Middle East respiratory syndrome (MERS-CoV) have both been documented as causing severe maternal and perinatal complications during pregnancy (Alfaraj et al., 2019; Wong et al., 2004). At this point there are sparse reports of COVID-19 in pregnant women. Chen et al. (2020) reported nine cases, all in their third trimester, from Wuhan in January 2020. All nine women delivered liveborn healthy infants via cesarean deliveries, with no significant complications. Amniotic fluid, cord blood, neonatal throat swabs, and milk samples from six of the mothers were tested for SARS-CoV-2, and all samples tested negative for the virus. We will come back to this finding.

## COVID-19 and Human Milk

Liu et al. (2020) identified 13 COVID-19 positive hospitalized pregnant women officially reported by the central government of China outside of Wuhan between December 8, 2019 and February 25, 2020. Three women improved and were discharged with ongoing pregnancies. The other 10 underwent cesarean deliveries because of complications, including preterm labor and one stillbirth. One mother developed multiple organ dysfunction syndrome, including acute respiratory distress syndrome (ARDS), and required extracorporeal membrane oxygenation (ECMO). At the time of publication of that article she was still on ECMO. The other nine mothers and eight liveborn infants were discharged home well. “There was no clinical or serologic evidence suggestive of vertical transmission of SARS-CoV-2” (Liu et al., 2020). But there was also no mention of evaluating the mothers’ milk.

What do we know about transmission of coronaviruses into human milk? During the SARS outbreak, a pregnant woman who contracted SARS-CoV in the second trimester and required mechanical ventilation ultimately recovered and delivered a healthy 38-week infant. At approximately 130 days after illness onset, antibodies to SARS-CoV were detected in maternal serum, cord blood, and milk with no evidence of virus (Robertson et al., 2004). As mentioned above, the finding of Chen et al. (2020) testing the milk from six postpartum women from China all were found negative for the SARS-CoV-2 virus. That is all that is currently known.

To understand effects on milk banking and donor milk, we also need to understand what is being reported about breastfeeding. Current recommendations in this pandemic from the Centers for Disease Control and Prevention (Centers for Disease Control and Prevention, 2020a) are given for mothers who give birth during the pandemic:

Infants born to mothers with confirmed COVID-19 should be considered “persons under investigation” (PUIs). As such, infants should be isolated.…To reduce the risk of transmission of the virus that causes COVID-19 from the mother to the newborn, facilities should consider temporarily separating (e.g., separate rooms) the mother who has confirmed COVID-19 or is a PUI from her baby until the mother’s transmission-based precautions are discontinued.…The risks and benefits of temporary separation of the mother from her baby should be discussed with the mother by the healthcare team… During temporary separation, mothers who intend to breastfeed should be encouraged to express their breast milk to establish and maintain milk supply. If possible, a dedicated breast pump should be provided. Prior to expressing breast milk, mothers should practice hand hygiene. After each pumping session, all parts that come into contact with breast milk should be thoroughly washed and the entire pump should be appropriately disinfected per the manufacturer’s instructions. This expressed breast milk should be fed to the newborn by a healthy caregiver. If a mother and newborn do room-in and the mother wishes to feed at the breast, she should put on a facemask and practice hand hygiene before each feeding (Centers for Disease Control and Prevention, 2020a).

More information can be found at Centers for Disease Control and Prevention (2020b)

WHO (2020c) and UNICEF agree, and differ from the CDC. UNICEF states:

Considering the benefits of breastfeeding and the insignificant role of breastmilk in the transmission of other respiratory viruses, the mother can continue breastfeeding, while applying all the necessary precautions. For symptomatic mothers well enough to breastfeed, this includes wearing a mask when near a child (including during feeding), washing hands before and after contact with the child (including feeding), and cleaning/disinfecting contaminated surfaces… (UNICEF, 2020).

WHO writes: “Infants born to mothers with suspected, probable or confirmed COVID-19 infection, should be fed according to standard infant feeding guidelines, while applying necessary precautions for IPC” [infection protection and control] (WHO, 2020c, p. 13). This is consistent with the *Global Strategy for Infant and Young Child Feeding* (WHO, 2003) in which all infants should initiate breastfeeding within one hour of birth, or if unable in that time frame, then be supported to do so as soon as able. In addition, they state:

symptomatic mothers who are breastfeeding or practicing skin-to-skin contact or kangaroo mother care should practice respiratory hygiene, including during feeding (for example, use of a medical mask when near a child if with respiratory symptoms), perform hand hygiene before and after contact with the child, and routinely clean and disinfect surfaces which the symptomatic mother has been in contact with (WHO, 2020c, p. 14).

Finally, concerning mothers who are too sick to directly breastfeed, “…mothers should be encouraged and supported to express milk, and safely provide breastmilk to the infant, while applying appropriate IPC measures (WHO, 2020c, p. 14).

## COVID-19 and Donor Milk Banking

How has this pandemic affected donor milk banking? One could envision, with the increasing numbers of confirmed COVID-19 cases, despite the relatively fewer numbers and less severe clinical courses of children affected so far (Dong et al., 2020), that the demand for donor milk would be at least as high as levels present before the onset of the pandemic. Through personal communications with colleagues in China, Italy, and my own donor milk bank in the United States, I have attempted to document what is currently happening.

I have been communicating for the past several days over social media with Professor Xihong Liu, Director of Clinical Nutrition, Department of Guangzhou Women and Children Medical Center, an affiliate of Guangzhou Medical University, Guangzhou, China. She founded the first donor milk bank in China. Partially in English and partially through a translator, (Virginia Chi Cheng Tam, IBCLC, *Macau Breastfeeding and Nurturing Promotion Association*, Macau, People’s Republic of China) Professor Liu has told me that both the supply and the demand of donor milk in China has decreased significantly with this infection. The milk banking processes were already quite strict prior to COVID-19, especially donor screening. However, screening has become “more careful and rigorous”. Prior to the outbreak, donors could express milk in their homes and deliver it to the hospitals (where milk banks are located). Technicians would also sometimes make milk pickups from donor homes. Both have now stopped. All milk is expressed in person. A donor’s temperature is taken, and she is required to fill out a questionnaire, including questions about whether she has herself been to or been in contact with anyone who has been to an epidemic area in the past 14 days. Almost every donor is asked to come to the hospital to donate, and all milk from home is refused. In some places, collections are made at the gates to communities from healthy, non-exposed donors. The containers are always supplied by the milk banks. As Professor Liu told me via text messaging on WhatsApp:

COVID-19 is affecting the world, [and is] also affecting the whole medical activity including donor milk banking. First, people are trying not to go outside, so [there are] fewer patients in the hospital, fewer patients come to the hospital, and fewer donors come to donate their milk. Of course, the demand and supply has been greatly reduced. We will decrease quantity but not quality. In the People’s Republic of China, milk banks only provide donor milk to hospitalized patients, not even for outpatient departments, let alone infants in society. The demand has also come down for milk, because less people venture to go to the hospitals. Even when people are sick, they stay away from the hospitals, assuming healthcare facilities are filled with COVID-19. Folks with other sicknesses do not stay in the hospitals as much during the outbreak, therefore both supply and demand of donor milk have decreased (Professor Xihong Liu, personal communication, March 17, 2020).

I have also been in contact with two colleagues in Italy, Dr. Enrico Bertino, Head of the Neonatal Unit of Turin University, City of Health and Science of Turin, Turin, Italy, and President of the *European Milk Bank Association* (EMBA), and Dr. Guido Moro, President of the *Italian Association of Human Milk Banks* (AIBLUD). When asked about the donor milk banking situation in Italy on March 16, they replied by email:

As you probably know, in Italy, we are forced to live in our houses without any possibility to go out (we are permitted to go out only for buying foods in supermarkets), and all the activities of our country are completely blocked. We do not know how long the health system and the economy of our country will be able to tolerate this situation. At the moment we are the only country in Europe with such extreme measures, but [we are] sure that in a couple of weeks also the other European country will be in a similar situation. Concerning your request, we can only tell you that donation of human milk has decreased a lot in this period: people cannot go out to bring the milk to the Banks, and even if theoretically they can be justified for their action of social value, mothers in this period prefer to stay as far as possible from hospitals. The other alternative is home milk collections from the banks, but also this activity has been reduced drastically because all the efforts of the hospitals are devoted to take care of people infected by COVID-19. In Milan we have a special system of home milk collection, called *Human Milk Link*, that is serving the three Human Milk Banks of the city of Milan. It is performed by a nurse, specialized in lactation, who is driving the car, collecting the milk and giving breastfeeding advices to the mothers. This service has been stopped exactly one week ago, Monday, March 9, 2020. So, donation of human milk in Milan has been practically suspended. This is the only information we can give you related to the situation of human milk donation and human milk banks in Italy in this period (personal communication, March 16, 2020).

I asked if an infant is in NICU and the mother is a person under investigations (PUI) or COVID positive, are they handling her expressed milk any differently? Are they decontaminating the outside of the containers her milk is expressed into, after she expresses it, before it is stored in the NICU refrigerators or freezers? They responded:

Indications in our country for PUI or COVID positive mothers who are expressing their milk are: they should utilize sterile containers (so no need for any specific treatment), should put on a facemask and practice a very careful hand hygiene before each feeding or other close contact with her newborn. The facemask should remain in place during contact with the newborn. These practices should continue while the mother is on transmission-based precautions in a healthcare facility (personal communication, March 16, 2020).

They sent me the document *Breastfeeding and SARS-CoV-2 Infection (Coronavirus Disease 2019—COVID-19*; see Supplemental materials) written by components of the Ministry of Health, Italian Society of Neonatology, and Italian Association of Human Milk Banks (2020), under which Italy is currently operating. It includes a table describing various scenarios of a mother’s health and an infant’s status, with breastfeeding or use of expressed milk. They are making every effort to ensure that mothers who can breastfeed do so and, if they cannot, that their milk is given to their infants.

I am the volunteer Co-Medical Director of the *Mother’s Milk Bank of the Western Great Lakes*, a Human Milk Banking Association of North America (HMBANA) non-profit milk bank located in Elk Grove Village, Illinois, USA (https://www.milkbankwgl.org/). I spoke by telephone with our Executive Director, Summer Kelly, MS, RN, IBCLC on March 16, about the effects coronavirus is having on our milk bank. She reported that we have not seen any effects yet on supply or demand. There is heightened anxiety in donors who must interact with the healthcare system to have their screening bloods drawn, or when they interact to drop off their milk at the milk bank, and with healthcare providers and recipients concerning the provision of the donor milk, i.e. “is it safe?”. We are currently engaged in educating mothers that we have no evidence of coronaviruses transmission through human milk, and that previous coronaviruses, although also not found in human milk, have been destroyed by pasteurization.

In complying with social distancing, this milk bank is sending staff home who can work from home. Should any staff develop COVID-19, we have put an emergency plan into place to stagger staffing between several groups—one group would work in the milk bank to pasteurize for 2 weeks; the milk bank would be deep cleaned, then rotate to another group. For emergency preparedness we have ordered extra supplies. As face masks are in short supply in the United States, and we do not wear them to protect ourselves but to protect the milk, one of our staff members is sewing masks that we can launder daily if we run out of disposables. If we cannot use our delivery services for the milk (United Parcel Service, FedEx), then we will deliver the milk ourselves. We cover two states and, from our milk bank, that is a 6-hour drive in each direction. We have increased production, so we have pasteurized milk as opposed to raw milk in our freezers should the donor supply dwindle. We are reaching out to our donors now to request more milk before the situation worsens here. We use milk depots throughout our catchment areas to which donor mothers can deliver their milk close to home, and from which it is shipped to us. Should these be closed due to government orders to enforce social distancing, as long as shipping options remain, we can still receive milk from donors. We use Styrofoam coolers and Techni Ice reusable dry ice packs, so we do not even need to have access to dry ice.

There have been official statements from milk banking organizations. EMBA issued its COVID-19 statement on February 25. It upholds the importance of donor milk, the fact that it is still unknown if SARS CoV-2 can be found in human milk, and, that if it is, it will likely be destroyed by pasteurization. They recommend adding questions to donor screening concerning risk of exposure, temporarily suspending recruitment of new donors and not accepting donations from current donors who may have been exposed, for 2 weeks. An established donor who develops symptoms should suspend donation and be tested. If the culture is positive for SARS CoV-2, donation should be interrupted until a negative culture is found. If the culture is negative for SARS CoV-2, donation can be continued (European Milk Banking Association, 2020).

HMBANA issued its guidance on March 6. It reiterated the similarities to SARS and MERS viruses, which have been shown to be completely inactivated by Holder pasteurization, the method used by all HMBANA milk banks, and that SARS was not found in any human milk. Donors are “screened regarding international travel as well as recent illness history including family members in the home. Mothers are deferred based on responses” (Human Milk Banking Association of North America, 2020). Amy Manning Vickers, MSN, RN, IBCLC, Executive Director of *Mothers’ Milk Bank of North Texas*, Fort Worth, Texas, and President of the *Human Milk Banking Association of North America* wrote:

As we all are, I am very concerned about how this crisis will unfold and impact nonprofit milk banking. It is very complex. I expect to see the supply of raw milk decrease as donors choose not to leave their homes and are reluctant [to] go into facilities to be lab tested. In anticipation of supply issues, we (my milk bank) have reached out to our recipient hospitals and asked them to more carefully prioritize the use of DHM [donor human milk] to the most vulnerable. We are also asking them to help us in identifying potential donors. We are also putting systems in place to pick up milk from donors safely without contact and are asking our depot sites to help by offering curbside drop off. We have sent a call to action to our current donors letting them know that donations are declining and that they can make an even bigger difference right now (personal communication, March 16, 2020).

On March 16, HMBANA sent a letter to its Executive Directors (personal communication, Summer Kelly, March 16, 2020). In it, the Guidelines Committee recommended a 28-day donating deferral for COVID-19 positive mothers, based on the US Food and Drug Administration advice to blood banks (U.S. Food and Drug Administration, 2020). This is not a requirement at this time, but a recommendation.

## Conclusion

Where are we today with donor milk banking, as I finish writing, but the pandemic is swelling around us, showing signs of slowing where it began in China, but still increasing in many places in the world? Donor milk banking is predicated always on the *protection, promotion and support of breastfeeding*. We believe this deadly virus is not transmitted through mother’s milk. We do not know if there is vertical transmission or not. As of this writing we are beginning to hear reports that the youngest among us may be a high-risk group, as they are with other viruses. As this is a novel virus, we, as a community, have not previously been exposed to it. There may not be specific immune factors against it in human milk, yet. But, as we know, there are so many immune factors in, and immune functions of, human milk, that its provision to our youngest and very vulnerable must continue to be of paramount importance. We know of one infected mother who had anti-SARS-1 antibody in her milk postpartum (Robertson, et al., 2004). The ability to provide support and to protect our breastfeeding mothers becomes extremely difficult where the virus is rampant. With social distancing, how do we provide the care, support, and protection these mothers need? In my discussions with my colleagues in Italy and China I heard the angst in their voices as they lamented their inability to see mothers and babies and provide this most basic of supports. If we cannot support breastfeeding, how will we ensure a supply of donor milk? In populations with large areas under quarantine or “lockdown”, how are we to move “safe” milk from the donors to the milk banks? In the bigger picture, what does this mean for the health of our children, as we may in fact see breastfeeding rates, and the supply and use of donor milk, decrease during this very unstable and concerning of times.

## Supplemental Material

Supplementary Material 1 - Supplemental material for International Perspectives Concerning Donor Milk Banking During the SARS-CoV-2 (COVID-19) Pandemic Click here for additional data file. Supplemental material, Supplementary Material 1, for International Perspectives Concerning Donor Milk Banking During the SARS-CoV-2 (COVID-19) Pandemic by and Kathleen A. Marinelli in Journal of Human Lactation
