# Supplementary material for: International Perspectives Concerning Donor Milk Banking During the SARS-CoV-2 (COVID-19) Pandemic
Source: J Hum Lact. 2020 Mar 30:0890334420917661. doi: 10.1177/0890334420917661 (PMC7201197; doi:10.1177/0890334420917661)
Supplement: Supplementary Material 1 - Supplemental material for International Perspectives Concerning Donor Milk Banking During the SARS-CoV-2 (COVID-19) Pandemic [file 10.1177_0890334420917661-suppl1.pdf]

28 February 2020

This document has been drawn up by

and endorsed by

BREASTFEEDING and SARS-CoV-2 INFECTION
(Coronavirus Disease 2019 - COVID-19)

Ad interim directions of the Italian Society of Neonatology (SIN)

Authors

This document has been drafted by Dr Riccardo Davanzo, Chair of the Technical Panel on Breastfeeding (TAS) of the Ministry of Health (MOH), and Prof. Fabio Mosca, President of the Italian Society of Neonatology (SIN), with the collaboration of Dr Guido Moro, President of AIBLUD (Human Milk Banking Association of Italy), Dr Fabrizio Sandri, Secretary of SIN and Prof. Massimo Agosti, President of the SIN Breastfeeding Commission.

Notice

Please note that information about COVID-19 transmission is emerging daily. This document has been prepared taking into account the scientific data available as of February 27, 2020. The guidance given is subject to change in the future with the acquisition of further knowledge about the SARS-CoV-2 epidemic, its perinatal transmission and clinical characteristics of cases of neonatal SARS-CoV-2 infection.

Glossary

We would like to clarify the meanings given to the terms SARS-CoV-2 and COVID-19 in this document.
The term SARS-CoV-2 is taken to mean the viral causative agent responsible for SARS, also called novel Wuhan nCoV-2019 coronavirus. The term SARS is the acronym for Severe Acute Respiratory Syndrome caused by coronavirus 2.
COVID-19 (Coronavirus Disease-2019) means the SARS-CoV-2 disease identified at the end of 2019 in the Wuhan Region, China.

Introduction

The recent epidemic caused by a novel coronavirus isolated in Wuhan (China) at the end of 2019 (SARS-CoV-2) raises, among other clinical and public health issues, questions relating to infection during pregnancy and the possible transmission of the infection from mother to child before, during and after childbirth. Doubts also arise as to the safety of the joint management of mother and child after childbirth and of breastfeeding. These questions are dealt with in this document.

Limits of current knowledge

- The SARS-CoV-2 virus spreads mainly from person to person through close contact (0-2 metres), and is transmitted by means of respiratory secretions (droplets) when an infected person sneezes or coughs.

- It is not yet clear what possible impact a perinatal transmission of SARS-CoV-2 infection might have, the outcome of which, in a similar way to past epidemics SARS-CoV-1 (Severe Acute Respiratory Syndrome) and MERS-CoV (Middle East Respiratory Syndrome Coronavirus), may depend more on the severity of the maternal infection and on concomitant obstetric pathologies than on the SARS-CoV-2 infection itself.

- A possible neonatal SARS-CoV-2 infection might be the result of a transmission acquired by the mother via the respiratory route in the postpartum period rather than transplacentally.

The possibility of respiratory infections from common coronaviruses in the neonatal period and in the first year of life was already known before the current SARS-CoV-2 outbreak. A cohort of children whose mothers had suspected COVID-19, with respiratory symptoms appearing in the first week of life and a clinical diagnosis of pneumonia, has been described by Zhu (2020), but Real Time PCR testing of viral RNA from neonates' pharyngeal swabs proved to be negative, thus did not corroborate the SARS-CoV-2 aetiology. Based on the few data available in literature, postnatal SARS-CoV-2 infection would appear to be not severe or even asymptomatic compared to what occurs in older age groups.

Protection of breastfeeding integrated with sanitation measures

- Breastfeeding and the use of breast milk provides protection for both mother and child, and has additional family, social and economic benefits.

- In the event of maternal SARS-CoV-2 infection, breast milk is not considered to be a transmission vehicle, based on current scientific knowledge and in a similar way to other known respiratory viral infections.

- Nevertheless, the current SARS-CoV-2 epidemic requires us to combine the promotion of breastfeeding with a correct health and hygiene approach, limiting the contagion by air and by contact with the respiratory secretions of infected patients (including mothers having just given birth).

- It is likely, although not yet documented, that in breast fed infants specific SARS-CoV-2 antibodies can pass from the mother with COVID-19 to the infant within a few days of onset of the disease, modulating the clinical expression of the infant's infection.

- An approach involving the routine separation of the newborn from the SARS-CoV-2 positive mother may have an adverse effect on the mother-child relationship, and may represent a belated intervention to prevent a contagion already occurred in the pre-symptomatic phase.

Currently available directions on the prevention of transmission from mother to child

- Chinese Paediatrics COVID-19 Working Group. Doctors (Wang 2020, Quiao 2020) who recently have dealt with the COVID-19 epidemic in China suggest feeding with infant formula or possibly donor breast milk. The authors do not provide specific reasons for this choice. In the recommendation of the Chinese experts, presumably umbalanced on the side of caution, there is no reference to an overall assessment of the risks of infection compared with those of not allowing breastfeeding.

- UNICEF. This Agency does not consider the option of separating mother and child, and suggests continuing breastfeeding, with the parallel adoption of hygiene measures to reduce the possible transmission from mother to child of the SARS-CoV-2 infection.

- Centers for Disease Control (CDC). According to the CDC (2020):

    - If the mother is a person under investigation or tests positive for SARS-CoV-2, the option of separate management of mother and child should be considered as the first choice. The risks and benefits of this separation and the consequences of not starting, continuing or suspending breastfeeding should be shared with the family and with healthcare professionals.

    - If the mother and child are managed jointly and the mother breastfeeds her child, the usual measures aimed at preventing the transmission of the viral infection should be put in place: avoid kissing the child, protect him from adult coughing and respiratory secretions (wear a mask during feeding and intimate contact with the baby), wash hands, in particular before feeding, suspend visits.

    - If the child remains in hospital with the mother in a rooming-in regimen, he will be made to sleep in his cradle at a distance of at least 2 metres from the mother.

- Italian National Institute of Health (Istituto Superiore di Sanità; ISS). In view of currently available scientific information and the protective potential of breast milk, it is believed that, in the case of a woman with suspected SARS-CoV-2 infection or with COVID-19, under clinical conditions that allow it and according to her desire, breastfeeding should be started and/or continued directly from the breast or using expressed breast milk. To reduce the risk of transmission to the child, preventive procedures such as hand hygiene and the use of a face mask during feeds are advisable, according to the recommendations of the Italian Ministry of Health (MOH).

Should the mother and child be temporarily separated, it is recommended that the mother be helped to maintain milk production through manual or mechanical expression, following the same rules of hygiene. For all healthcare workers and professionals who come into contact with pregnant women and newborn infants, preventive recommendations should be observed.

Use of expressed breast milk

- In case of separation of mother and infant, the automatic use of breast milk substitutes should be avoided. Mothers who intend to breastfeed / continue breasfeeding should be encouraged to express their breast milk to establish and maintain milk supply.

- Expressed breast milk should not be pasteurised before being given to the baby, as it is believed that, even if containing SARS-CoV-2, it would not be a vehicle of infection.

- The use of expressed milk from a SARS-CoV-2 positive mother in Neonatal Intensive Care Units (NICUs) follows specific protocols.

- In cases of severe maternal infection, expressing breast milk can be inappropriate and should be avoided due to the mother's general conditions

Final provisional directions on the management of mother and newborn during the SARS-CoV-2 epidemic.

The directions are summarised in table 1.

- Whenever possible, the preferred option is the joint management of mother and child, in order to facilitate interaction and the beginning of breastfeeding. This choice is feasible when a mother previously identified as SARS-CoV-2 positive is asymptomatic or paucisymptomatic or on the way to recovery, or when a mother that is asymptomatic or paucisymptomatic is under investigation for SARS-CoV-2.

- If the mother has a severely symptomatic respiratory infection (with fever, coughing and respiratory secretions), mother and child should be temporarily separated, pending the response of the laboratory test (RNA-PCR) for coronavirus. If the test is positive, mother and child continue to be managed separately; if the test is negative, rooming-in for mother and child is applicable.

- The decision about whether or not to separate mother and child must be taken on an individual basis, taking into account the informed consent of the mother, the hospital logistics and possibly the local epidemiological situation relating to the spread of SARS-CoV-2.

- If the infant is separated from the mother, the use of fresh expressed breast milk is recommended, while the pasteurisation of breast milk is not.

- In the case of a SARS-CoV-2 positive mother, strict hygiene measures should always be adopted to prevent the possible transmission of the infection by air or by contact with respiratory secretions. The newborn infant, other hospitalised patients and healthcare personnel should therefore be protected.

- The compatibility of breastfeeding with drugs that may be administered to women with COVID-19 should be assessed on a case-by-case basis.

Table 1. Directions on mother-infant management in the perinatal period.

State of the mother	RNA-PCR testing on the mother for SARS-CoV-2 using pharyngeal swab	RNA-PCR testing on the newborn for SARS-CoV-2 using pharyngeal swab	Isolation of the mother°	Management of the newborn during hospital stay°	Breastfeeding advice	Preventive measures for mother-child transmission §
Asymptomatic or paucisymptomatic mother known to be SARS-CoV-2 positive	Already done	YES	YES, in a dedicated area of postpartum ward	In a rooming-in regimen, but in an isolated and dedicated area of postpartum ward	YES	YES
SARS-CoV-2 paucisymptomatic mother under investigation	Yes	Only if maternal test is positive	YES, in a isolated and dedicated area of postpartum ward, pending result of the lab test	In a rooming-in regimen, but in a isolated and dedicated area of postpartum ward, at least until the result of the lab test	YES	YES
Mother with respiratory infection symptoms (fever, cough, secretions) with a positive SARS-CoV-2 or under investigation	YES or already being done	Only if maternal test is positive	YES, in a dedicated area of postpartum ward, pending result of the lab test	Newborn isolated and separated from the mother, at least until the result of the lab test.<br>He is placed in a dedicated and isolated area in the Neonatology Unit (if asymptomatic) or in the NICU (if affected by respiratory disease)	NO; use of expressed milk.^ Pasteurisation is not recommended	YES

§ Room divider or curtain, surgical face mask for the mother when breastfeeding or in intimate contact with the newborn, careful washing of hands, placing the baby's cradle at a distance of 2 metres from the mother's head, no visits from relatives and friends.

° In addition, adequate protection measures for healthcare personnel, according to the indication of the MOH of Italy

^ Mother's fresh milk should be extracted with a manual or electrical breast pump. The mother should always wash her hands before touching bottles and all breast pump parts, following recommendations for proper washing of the breast pump after each use.

References

1. Davanzo R., Romagnoli C, Corsello G. Position Statement on Breastfeeding from the Italian Pediatric Societies. Italian Journal of pediatrics 2015 (41) 80: 1-3

2. Emergency response plan for the neonatal intensive care unit during epidemic of 2019 novel coronavirus. Zhongguo Dang Dai Er Ke Za Zhi. 2020 Feb;22(2):91-95.

3. Gagneur A, Dirson E, Audebert S, Vallet S, Quillien MC, Baron R, Laurent Y, Collet M, Sizun J, Oger E, Payan C [Vertical transmission of human coronavirus. Prospective pilot study]. Pathol Biol (Paris). 2007 Dec;55(10):525-30. Epub 2007 Sep 21.

4. https://www.cdc.gov/coronavirus/2019-ncov/hcp/inpatient-obstetric-healthcare-guidance.html

5. https://www.cdc.gov/coronavirus/2019-ncov/specific-groups/pregnancy-guidance-breastfeeding.html

6. https://www.dailymail.co.uk/news/article-8038035/17-day-old-baby-girl-recovers-coronavirus-without-medication.html

7. https://www.unicef.org.au/blog/news-and-insights/february-2020/novel-coronavirus-outbreak-what-parents-need-to-know

8. https://www.epicentro.iss.it/coronavirus/gravidanza-parto-allattamento

9. Li AM, Ng PC. Severe acute respiratory syndrome (SARS) in neonates and children. Arch Dis Child Fetal Neonatal Ed. 2005 Nov;90(6):F461-5.

10. Qiao J. What are the risks of COVID-19 infection in pregnant women? The Lancet. doi:10.1016/s0140-6736(20)30365-2

11. Shek CC, Ng PC, Fung GP, Cheng FW, Chan PK, Peiris MJ, Lee KH, Wong SF, Cheung HM, Li AM, Hon EK, Yeung CK, Chow CB, Tam JS, Chiu MC, Fok TF. Infants born to mothers with severe acute respiratory syndrome. Pediatrics. 2003 Oct;112(4):e254.

12. Wang L et al. Working Committee on Perinatal and Neonatal Management for the Prevention and Control of the 2019 Novel Coronavirus Infection. Chinese expert consensus on the perinatal and neonatal management for the prevention and control of the 2019 novel coronavirus infection (First Edition). Ann Transl Med 2020: 8 (3); 47

13. Working Group for the Prevention and Control of Neonatal 2019-nCoV Infection in the Perinatal Period of the Editorial Committee of Chinese Journal of Contemporary Pediatrics. Perinatal and neonatal management plan for prevention and control of 2019 novel coronavirus infection (1st Edition)]. Zhongguo Dang Dai Er Ke Za Zhi. 2020 Feb;22(2):87-90. Chinese.

14. Zhu H, Wang L, Fang C, et al. Clinical analysis of 10 neonates born to mothers with 2019-nCoV pneumonia. Transl pediatr 2020; 9 (1): 51-60
